# Arrest Defective-1 Controls Tumor Cell Behavior by Acetylating Myosin Light Chain Kinase

**DOI:** 10.1371/journal.pone.0007451

**Published:** 2009-10-14

**Authors:** Dong Hoon Shin, Yang-Sook Chun, Kyoung-Hwa Lee, Hyun-Woo Shin, Jong-Wan Park

**Affiliations:** 1 Department of Pharmacology, Ischemic/Hypoxic Disease Institute, Seoul National University College of Medicine, Chongno-gu, Seoul, Korea; 2 Department of Physiology, Ischemic/Hypoxic Disease Institute, Seoul National University College of Medicine, Chongno-gu, Seoul, Korea; Bauer Research Foundation, United States of America

## Abstract

**Background:**

The enhancement of cell motility is a critical event during tumor cell spreading. Since myosin light chain kinase (MLCK) regulates cell behavior, it is regarded as a promising target in terms of preventing tumor invasion and metastasis. Since MLCK was identified to be associated with human arrest defective-1 (hARD1) through yeast two-hybrid screening, we here tested the possibility that hARD1 acts as a regulator of MLCK and by so doing controls tumor cell motility.

**Methodology/Principal Findings:**

The physical interaction between MLCK and hARD1 was confirmed both *in vivo* and *in vitro* by immunoprecipitation assay and affinity chromatography. hARD1, which is known to have the activity of protein lysine ε-acetylation, bound to and acetylated MLCK activated by Ca^2+^ signaling, and by so doing deactivated MLCK, which led to a reduction in the phosphorylation of MLC. Furthermore, hARD1 inhibited tumor cell migration and invasion MLCK-dependently. Our mutation study revealed that hARD1 associated with an IgG motif of MLCK and acetylated the Lys608 residue in this motif. The K608A-mutated MLCK was neither acetylated nor inactivated by hARD1, and its stimulatory effect on cell motility was not inhibited by hARD1.

**Conclusion/Significance:**

These results indicate that hARD1 is a bona fide regulator of MLCK, and that hARD1 plays a crucial role in the balance between tumor cell migration and stasis. Thus, hARD1 could be a therapeutic target in the context of preventing tumor invasion and metastasis.

## Introduction

Despite substantial improvements in cancer therapy, tumor invasion and metastasis remain the main causes of cancer death. Tumor invasion and metastasis are complicated processes, which involve alterations to cell adhesion, the proteolysis of matrix proteins, increased cell motility, intra/extravasation, and colonization at distant sites [1]. Since tumor cell migration is a key step in tumor spread, to identify key regulators of cell motility might provide novel therapeutic possibilities. In this respect, myosin light chain kinase (MLCK) is regarded as a target for preventing tumor spread. Indeed, MLCK activation and expression have been found to be positively related with metastatic propensity [2], [3]. Moreover, MLCK inhibitors have been shown to diminish the invasiveness of various cancer cells [4], [5]. Therefore, it is worth while to understand the regulatory mechanism of MLCK in order to search for novel targets for preventing tumor spread.

MLCK is a Ca^2+^/calmodulin-dependent protein kinase that regulates a variety of cellular functions, such as, muscle contraction and cell migration. In mammals, MLCK proteins are encoded by two genes, namely, *mylk1* and *mylk2* [6], [7]. The *mylk1* gene is ubiquitously expressed in smooth muscle and non-muscle cells, whereas the *mylk2* is exclusively expressed in skeletal muscle cells [8]–[10]. The *mylk1* gene expresses at least four transcripts derived from independent promoters, namely, two long MLCKs, short MLCK, and telokin. Short MLCK (∼130 kDa) is expressed mainly in smooth muscle cells and long MLCK (∼210 kDa) is expressed in non-muscle cells [11]. Furthermore, long MLCK is expressed in several variant forms due to alternative mRNA splicing. Structurally, short MLCK has three immunoglobulin (Ig) motifs, one fibronectin-like (Fn) motif, a PEVK-rich repeat region, and three DFRXXL repeats in its N-terminus. Long MLCK is identical to short MLCK but has six additional Ig motifs and two additional DXRXXL motifs in its N-terminal extension region [12], [13].

From a mechanistic point of view, when cytosolic Ca^2+^ concentrations increase, Ca^2+^/calmodulin complex activates MLCK, which in turn phosphorylates the Thr18 and Ser19 residues in myosin light chain (MLC) [11], [14]. In addition, MLCK activity is finely regulated by the protein kinase network. Activated MLC is dephosphorylated and deactivated by protein phosphatase (PP)-IM [15], [16]. Furthermore, in addition to MLCK, Rho-kinase can phosphorylate MLC independently of Ca^2+^ and calmodulin [17]. Rho-kinase also phosphorylates PP-1M, which in turn inhibits the PP-IM-mediated dephosphorylation of phospho-MLC [18]. Finally, MLC phosphorylation stimulates myofilament assembly and myosin ATPase activity [19]. As a consequence, MLC phosphorylation enhances many contractile processes, such as, muscle contraction, stress fiber formation, focal adhesion, cell migration, and cytokinesis [6], [11].

Arrest defective-1 (ARD1) was first identified in *Saccharomyces cerevisiae* [20], and was subsequently found to act as an acetyltransferase and to regulate cell entry into the stationary phase and sporulation in yeast [21], [22]. Like yeast ARD1, mammalian homologues of ARD1 acetylate the N-terminal α-amino group of some proteins in association with N-acetyltransferase [23], [24]. In addition, mammalian ARD1s can acetylate the ε-amino group of lysine, for example, mouse ARD1 has been reported to acetylate Lys532 of HIF-1α and to promote HIF-1α ubiquitination and degradation [25], [26]. More recently, human ARD1 was reported to enhance lung cancer cell proliferation by acetylating and activating β-catenin [27]. Therefore, it is believed that ARD1 has diverse biological functions in mammalian cells, and that it acetylates various molecules in addition to HIF-1α and β-catenin.

In the present study, yeast two-hybrid assays showed that human ARD1 (hARD1) binds with long MLCK. Based on this interaction, we hypothesized that hARD1 acetylates long MLCK and that acetylated long MLCK affects tumor cell behaviors like migration and invasion. It was found that hARD1 binds to the N-terminal motif of long MLCK and acetylates a lysine residue within this motif. Furthermore, this hARD1-mediated acetylation was found to inactivate long MLCK, and thereby, to inhibit MLC phosphorylation, which in turn inhibited tumor cell migration and invasion. Based on these results, we propose that hARD1, an endogenous inhibitor of MLCK, plays a critical role in the control of tumor cell behavior, and suggest that hARD1 be viewed as a novel target molecule for the prevention of tumor invasion and metastasis.

## Results

### hARD1 associates with MLCK

**Figure 1:**
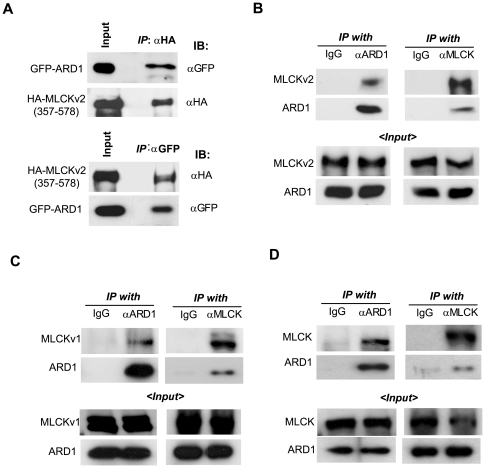
hARD1 associates with long MLCKs. (A–C) Interaction between expressed hARD1 and expressed long MLCKs. HT1080 cells were co-transfected with plasmids (4 µg each per 100 Φ dish) of GFP-ARD1 and MLCK variant 2 fragment (aa. 357–578) (A), ARD1 and full-length long MLCK variant 2 (MLCKv2) (B), or ARD1 and full-length long MLCK variant 1 (MLCKv1) (C). GFP-ARD1 or ARD1 was immunoprecipitated with anti-GFP, anti-ARD1, or non-immunized rat serum (IgG), and co-precipitated MLCKs were analyzed using anti-HA or anti-MLCK antibody. Otherwise, HA-MLCK or MLCK was immunoprecipitated with anti-HA, anti-MLCK, or non-immunized mouse serum (IgG), and co-precipitated ARD1 was identified using anti-GFP or anti-ARD1 antibody. (D) Interaction between endogenous hARD1 and long MLCK. In HT1080 cells, ARD1 was immunoprecipitated with anti-ARD1 or non-immunized serum, and co-precipitated MLCK was identified using anti-MLCK antibody (left panel). The interaction was also cross-checked by changing antibodies (right panel).

We screened a human liver cDNA library using the yeast two-hybrid method using full-length hARD1 as bait. Of twelve positive clones, three clones contained cDNA encoding aa. 357–578 of long MLCK transcript variant 2. We co-transfected HEK293 cells with GFP-ARD1 and HA-MLCK (357–578) plasmids and found that these peptides were co-precipitated (Fig. 1A). Since long MLCK variant 1 has 75 amino acids in addition to the entire sequence of variant 2, it was also expected to associate with hARD1. Both types of MLCK were co-precipitated with hARD1 (Figures 1B and 1C). These interactions were also crosschecked by changing the antibodies used for immunoprecipitation and immunoblotting. To identify the association between endogenous hARD1 and long MLCK, we chose HT1080 cell line which had been identified to express both hARD1 and long MLCK at high levels in a preliminary study. As expected, hARD1 and MLCK were shown to be naturally associated in HT1080 cells (Fig. 1D). Because long MLCK variant 1 is known to be more widely distributed than variant 2, we decided to study only long MLCK variant 1 in the following experiments and hereafter refer to it as MLCK.

### hARD1 binds and acetylates MLCK activated by Ca^2+^/calmodulin

**Figure 2:**
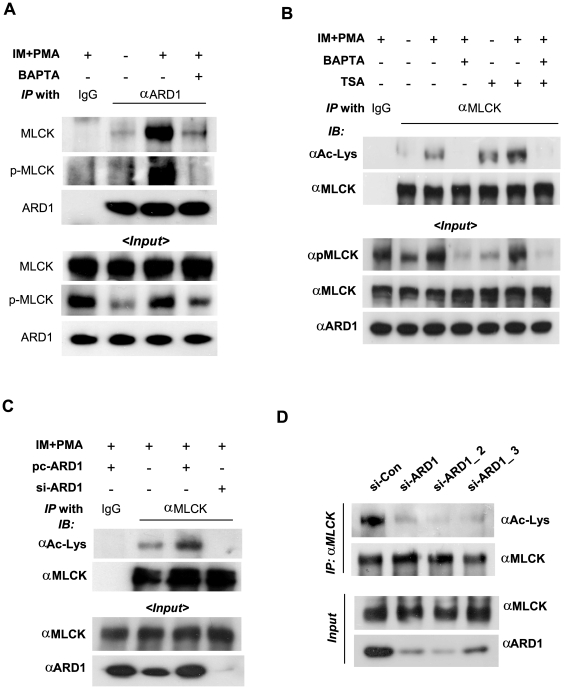
hARD1-binding and lysyl-acetylation of MLCK are stimulated by Ca^2+^ signaling. (A) The hARD1-MLCK interaction is stimulated by the Ca^2+^ signaling. HT1080 cells were co-transfected with 4 µg each of pcARD1 and pcMLCK. The cells were treated with 1 µM ionomycin (IM) and 30 nM phorbol myristate acetate (PMA) for 8 h, or 20 µM BAPTA-AM for 3 h. MLCK was co-immunoprecipitated by anti-ARD1 antibody. Co-precipitated proteins were analyzed by immunoblotting (upper panel). The input levels were analyzed by immunoblotting (lower panel). (B) The lysyl-acetylation of MLCK is augmented by Ca^2+^ signaling. Untransfected HT1080 cells were treated with ionomycin and PMA for 8 h or BAPTA-AM for 3 h, alternatively, Trichostatin-A (TSA) at 500 nM was treated for 5 h before cell harvest. The cell lysates were immunoprecipitated with anti-MLCK antibody, and immunoblotted by anti-acetyl-lysine or anti-MLCK antibody. (C) MLCK is acetylated by hARD1. HT1080 cells were transfected with pcARD1 (4 µg) or ARD1-silencing RNA (si-ARD1, 80 nM), and then treated with ionomycin and PMA for 8 h. In the samples immunoprecipitated with anti-MLCK antibody, total MLCK and acetylated MLCK were analyzed by immunoblotting. (D) Endogenous MLCK is acetylated hARD1-dependently. Endogenous MLCK was down-regulated in HT1080 cells using each of three siRNAs (80 nM) targeting different sites of ARD1 mRNA, and then treated with ionomycin and PMA for 8 h. Acetylated MLCK was immunoprecipitated by anti-MLCK antibody and immunoblotted with anti-acetyl-lysine antibody. Input protein levels were analyzed by immuboblotting with specific antibodies.

MLCK is phosphorylated and activated by Ca^2+^ signaling [6], [11]. To examine whether the MLCK/hARD1 interaction depends on MLCK activation, HT1080 cells were treated with ionomycin and PMA, which are known to activate MLCK by inducing Ca^2+^ influx and MLCK phophorylation, alternatively, cells were treated with BAPTA-AM to remove intracellular Ca^2+^. It is shown in Figure S1 that these reagents at the concentrations used did not affect cell viability. The hARD1/MLCK interaction was robustly stimulated by Ca^2+^ influx, which was abolished by Ca^2+^ chelation (Fig. 2A). This suggests that hARD1 primarily interacts with Ca^2+^-activated MLCK rather than inactive MLCK. Also, the MLCK was phosphorylated at Ser439 Ca^2+^-dependently and the phosphorylated MLCK was co-precipitated with hARD1 (Fig. 2A). Therefore, it is also possible that the MLCK/hARD1 interaction is affected by the MLCK phosphorylatin. We next tested the possibility that hARD1 acetylates the lysine residues of MLCK. The lysyl-acetylation of MLCK was stimulated by ionomycin/PMA and inhibited by BAPTA-AM, which was also associated with the phosphorylation of MLCK (Fig. 2B). In addition, the acetylated MLCK levels increased in the presence of TSA (a deacetylase inhibitor), which indicates that this acetylation can be reversed by some deacetylase. The Ca^2+^-mediated MLCK acetylation was further increased by hARD1 expression and abolished by hARD1 knock-down (Fig. 2C). To examine whether hARD1 naturally acetylates MLCK, we suppressed the expression of endogenous hARD1 using three siRNAs targeting different sequences of hARD mRNA. These siRNAs down-regulated hARD1, and also noticeably reduced the cellular levels of acetylated MLCK. Taken together, hARD1 may function to lysyl-acetylate endogenous and expressed MLCK.

### hARD1 inhibits the phosphorylation of MLC by inactivating MLCK

**Figure 3:**
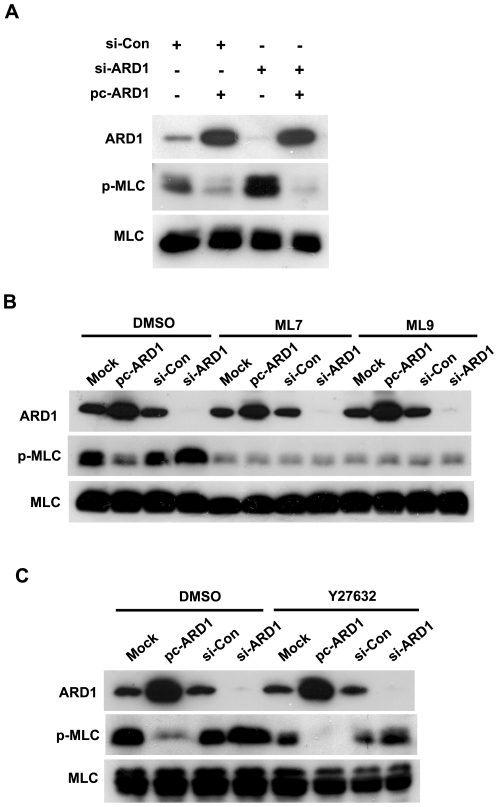
hARD1 inhibits the MLCK-dependent MLC phosphorylation. (A) hARD1 negatively regulates the phosphorylation of MLC. After HT1080 cells were transfected with 80 nM of ARD1 siRNA (si-ARD1) and/or 2 µg of pcARD1, Western blot analyses were performed. (B–C) hARD1 inhibits the phosphorylation of MLC MLCK-dependently but not Rho-kinase-independently. After transfected with 2 µg of phARD1 and/or 80 nM of siARD1, HT1080 cells were treated for 16 h with 20 µM of MLCK inhibitors (ML-7 and ML-9, B) or with 10 µM of a Rho-kinase inhibitor (Y27632, C), and then immunoblotted. DMSO was used as a vehicle control.

To understand the consequences of MLCK acetylation, we analyzed the phosphorylation of MLC in HT1080 cells transfected with hARD1 plasmid or siRNA. Phospho-MLC levels were found to be noticeably reduced by hARD1 overexpression, and increased by hARD1 knock-down (Fig. 3A). These results suggest that MLCK is functionally inhibited by hARD1. Furthermore, because MLC can be phosphorylated by either MLCK or Rho-kinase, we sought to indentify the kinase linked with hARD1-dependent MLC dephosphorylation. In HT1080 cells treated with DMSO-vehicle only, phospho-MLC levels were found to be inversely correlated with hARD1 expression (Figs. 3B and 3C, the DMSO group). MLCK inhibitors ML7 and ML9 reduced phospho-MLC levels in all groups tested and more importantly, the effects of hARD1 on MLC phosphorylation were abolished by MLCK inhibition (Fig. 3B). When cells were treated with a Rho-kinase inhibitor Y27632, MLC phosphorylation was also partially reduced, but this phosphorylation was still regulated hARD1-dependently (Fig. 3C). These inhibitors used were confirmed not to affect cell viability (Figure S1). These findings indicate that although MLC phosphorylation is mediated by both MLCK and Rho-kinase in HT1080 cells, only MLCK-mediated MLC phosphorylation is likely to be regulated by hARD1.

### hARD1 inhibits cell migration and invasion

**Figure 4:**
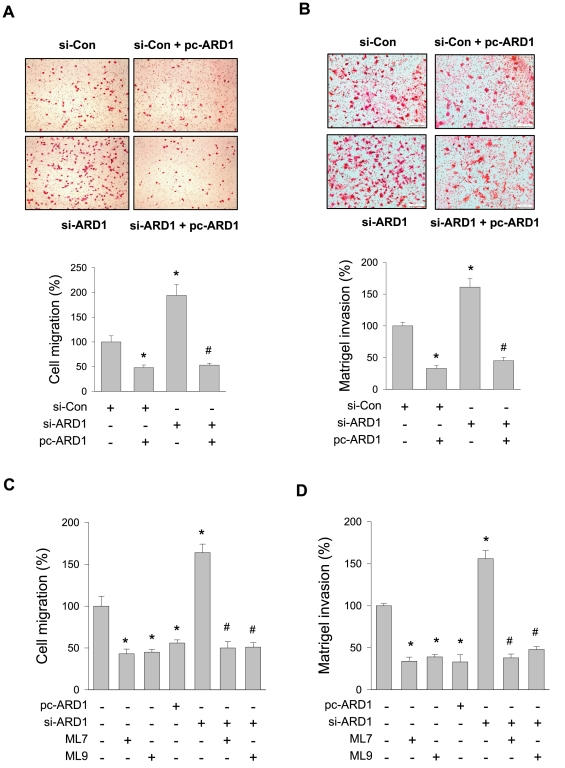
hARD1 inhibits migration and invasion of HT1080 cells. (A) Effect of hARD1 on cell migration. HT1080 cells were transfected with 80 nM of siARD1 and/or 2 µg of pcARD1. Cell migration was analyzed for 6 h in transwell chambers with a collagen-coated filter. Cells that had passed through the filter were stained with haematoxylin and eosin (upper panel). To quantify migrated cells, the stained cells were counted under a microscope at 40× magnification (lower panel). (B) Effect of hARD1 on cell invasion. HT1080 cells were seeded into the upper chamber with a filter double-coated with Matrigel in the upside and collagen in the downside. After incubated for 16 h, cells that had passed through the filter were stained (upper panel) and counted (lower panel). (C–D) ARD1 controls cell migration and invasion via MLCK. HT1080 cells were transfected with pcARD1 or si-ARD1, and then treated with 20 µM of ML-7 or ML-9 for 16 h. Cell migration and invasion were performed as mentioned above. *, *p*<0.001 versus the control group (*n* = 6); #, *p*<0.0001 versus the si-ARD1 group (*n* = 6).

Since the MLCK-MLC pathway is known to regulate cell contraction and migration, we investigated if hARD1 controls tumor cell migration and invasion by inhibiting this pathway. hARD1 overexpression was found to reduce the number of migrating cells by 52%, but hARD1 knock-down enhanced cell migration by 94%, and this enhancement was attenuated by hARD1 overexpression (Fig. 4A). Invasion assays also demonstrated that cell invasion was negatively regulated by hARD1 (Fig. 4B). These results suggest that hARD1 appears to function as a negative regulator of tumor cell migration and invasion.

### hARD1 controls cell behavior by inactivating MLCK

To examine whether hARD1 inhibits cell motility via MLCK, transfected HT1080 cells were treated with ML7 or ML9. The MLCK inhibitors significantly inhibited the cell migration and invasion, and also abolished the stimulatory effect of hARD1 knock-down on the cell motility. However, the MLCK inhibitors did not further inhibit the cell migration and invasion below the levels in hARD1-overexpressing cells (Fig. 4C and Fig. 4D). These results suggest that the action of hARD1 is due to its inactivation of MLCK. Furthermore, because stress fiber and actin filament formations are regulated by MLCK, we examined how cytoskeletal organization is regulated by hARD1. Filamentous actin (F-actin) of HT1080 cells treated with ML7 or ML9 was thinner than in untreated control cells, and a similar finding was observed in hARD1 overexpressing cells. Conversely, F-actin was thicker in hARD1 knocked-down cells, but became thinner by treatment of MLCK inhibitors (Figure S2). These results support the notion that hARD1 inhibits tumor cell migration and invasion by inhibiting MLCK. However, since both ML-7 and ML-9 could target molecules other than MLCK, it should be noted that the MLCK dependent action of hARD1 remains to be further investigated.

### Identification of the hARD1-binding domain of MLCK

**Figure 5:**
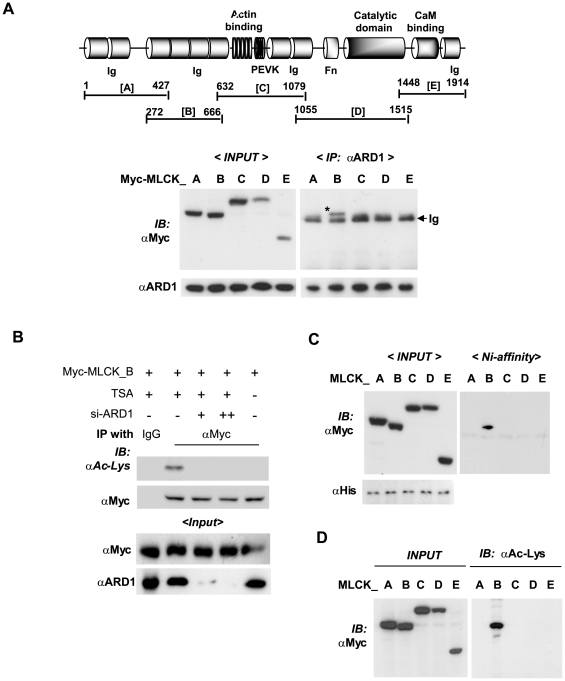
Identification of the hARD1-binding domain in long MLCK. (A) *In vivo* binding of hARD1 to five fragments of MLCK. HEK293 cells were co-transfected with 4 µg each of pcARD1 and one of pcMyc-MLCK fragments (shown in the upper panel). To examine which region of MLCK is associated with hARD1, immunoprecipitation was performed with anti-ARD1 antibody and then immunoblotted with anti-Myc antibody (right panel). The input levels were analyzed by immunoblotting (left panel). An asterisk and an arrow indicate the hARD1-coprecipitated protein band and the immunoglobulin heavy chain band, respectively. (B) *In vivo* acetylation of Myc-MLCK_B by hARD1. HT1080 cells were transfected with pcMyc-MLCK_B (4 µg) and/or si-ARD1 (40 nM or 80 nM). Trichostatin-A (TSA) at 500 nM was treated for 5 h before cell harvest. After immunoprecipitation with anti-Myc antibody, the lysyl-acetylation of Myc-MLCK_B was identified using anti-acetyl-lysine antibody. (C) *In vitro* binding of hARD1 to five fragments of MLCK. The extracted Myc-MLCK peptides were incubated with purified recombinant His-ARD1 at 4°C for 2 h. His-ARD1 was pulled-down using nickel-affinity beads, and then co-purified Myc-MLCK peptides were identified using anti-Myc antibody. (D) *In vitro* acetylation of Myc-MLCK peptides by hARD1. Purified His-ARD1 and Myc-MLCK peptides were incubated in the acetyl-CoA-containing reaction mixture for protein acetylation at 32°C for 4 h. Acetylated MLCK peptides were identified using anti-acetyl-lysine antibody.

To identify the region of MLCK targeted by hARD1, we co-transfected HEK293 cells with hARD1 plasmid and each of the five Myc-tagged plasmids expressing different MLCK segments. hARD1 was precipitated with anti-ARD1 antibody and was found to associate with MLCK_B of the N-terminal extension region (Fig. 5A). Furthermore, MLCK_B was found to be specifically acetylated by hARD1 in cells (Fig. 5B). Moreover, when TSA was omitted from culture medium, acetylated MLCK_B levels were significantly reduced. To examine the interaction between hARD1 and MLCK_B further, we mixed *in vitro* His-ARD1 with each of the five Myc-MLCK fragments. The purity (>97%) of His-hARD1 used was assessed by protein staining (Figure S3). After protein mixtures were pulled-down with Ni-NTA beads, immunoblotting was conducted with anti-Myc antibody. Of the five fragments, only MLCK_B was found to directly bind His-ARD1 (Fig. 5C). Next, we performed an *in vitro* acetylation assay, and of the five fragments, only MLCK_B was acetylated by hARD1 (Fig. 5D). These results suggest that hARD1 targets and acetylates a lysine within the aa.272–666 of MLCK.

### Lys608 acetylation of MLCK is responsible for hARD1 control of cell behavior

**Figure 6:**
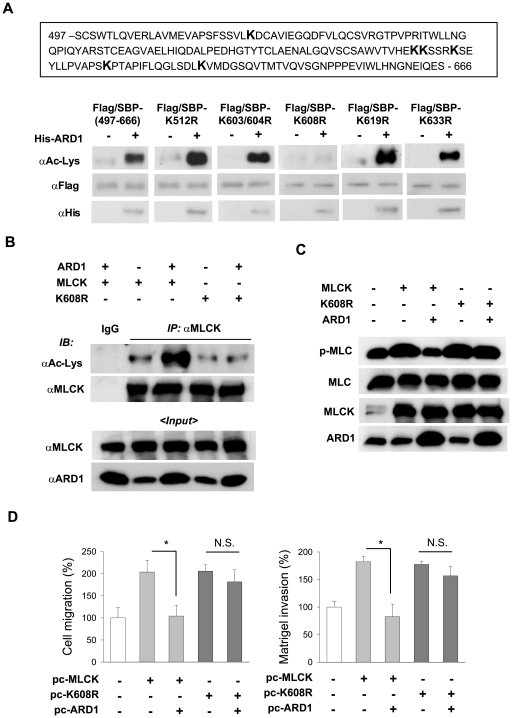
Identification of the acetylation site in long MLCK. (A) *In vitro* acetylation of MLCK peptides point-mutated at a lysine residue. The amino acid sequence of expressed MLCK(497–666) peptide is presented in the upper panel. Lysine residue is marked as K in bold font. Wild-type and mutated peptides were expressed in HEK293 and purified using Flag-affinity chromatography. The peptides and recombinant His-ARD1 protein were incubated in the reaction mixture for acetylation at 32°C for 4 h, and then lysyl-acetylated peptides were identified using anti-acetyl-lysine antibody (lower panel). (B) *In vivo* acetylation of full-length MLCK(K608R) mutant by hARD1. HT1080 cells were co-transfected with 4 µg of pcMLCK or pcMLCK(K608R) and/or 4 µg of pcARD1, and treated with 1 µM ionomycin, 30 nM PMA, and 0.5 µM TSA for 5 h before cell harvest. After immunoprecipitation with anti-MLCK antibody, the lysyl-acetylation of MLCK was identified using anti-acetyl-lysine antibody. (C) Lys608 of MLCK is required for hARD1 to inhibit the MLCK activity on MLC phosphorylation. HT1080 cells were co-transfected with 2 µg of pcMLCK or pcMLCK(K608R) and/or 2 µg of pcARD1. Cell lysates were immunoblotted by anti-phospho-MLC, anti-MLC, anti-MLCK, and anti-ARD1 antibodies. (D) Lys608 of MLCK is required for hARD1 to control cell motility. HT1080 cells were co-transfected with 2 µg of pcMLCK or pcMLCK(K608R) and/or 2 µg of pcARD1. Cell migration (left panel) and invasion (right panel) were analyzed in the transwell system. Migrating cells were stained with haematoxylin and eosin, and counted at 40× magnification. *, *p*<0.0001 versus the MLCK group (*n* = 6); N.S., not significant statistically (*n* = 6).

To identify the lysine residue acetylated by hARD1, we constructed a Flag/SBP-tagged recombinant protein (aa.497–666), which did not contain the overlap region in MLCK_A and B and the 75 amino acid region missing in MLCK variant 2. Purified His-ARD1 and Flag/SBP-(497–666) were mixed with acetyl-CoA in the acetylation reaction buffer. The purities of both peptides were >97% (Figure S3). We also confirmed that Flag/SBP-(497–666) was acetylated by His-ARD1 *in vitro* (Fig. 6A, the 1^st^ column). MLCK has six lysine residues in its aa. 497 to 666 region, namely, K512, K603, K604, K608, K619, and K633. To identify the lysine residue acetylated by hARD1, we substituted each of these six lysines with arginine to produce five mutants. As shown in Fig. 6A, Flag-SBP-K608R peptide alone was not acetylated by His-ARD1. To determine whether Lys608 was the main site acetylated by hARD1, we constructed the full-length K608R mutant and transfected it into HT1080 cells. As previously demonstrated, the acetylation of wild-type MLCK was found to be markedly increased by hARD1 expression. However, K608R mutant was not further acetylated by hARD1 (Fig. 6B), indicating that hARD1 specifically acetylated the Lys608 residue in an IgG domain of MLCK. We next examined whether Lys608 of MLCK is required for hARD1 to inhibit MLC activity and tumor cell behavior. The full-length K608R stimulated the phosphorylation of MLC in the same manner as wild-type MLCK. However, this effect of K608R was not significantly affected by hARD1 expression (Fig. 6C). Likewise, the effects of K608R on cell migration and invasion were not significantly attenuated by hARD1 expression (Fig. 6D). These findings indicate that the hARD1-mediated Lys608 acetylation of MLCK is an important regulator of tumor cell behavior.

## Discussion

**Figure 7:**
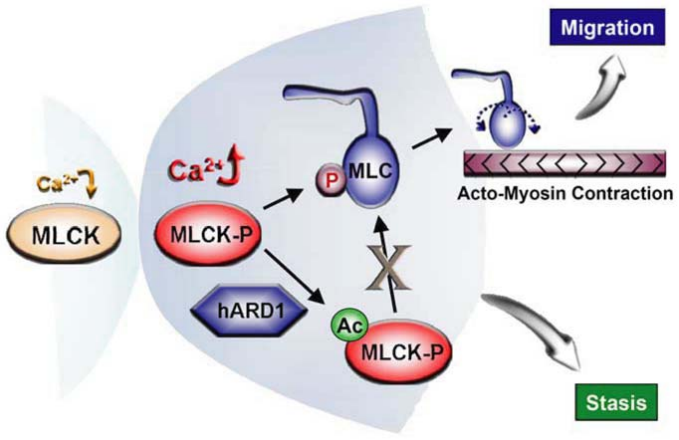
A schematic diagram summarizing cell motility regulation by MLCK and hARD1. Long MLCK is activated by the Ca^2+^ signaling, which phosphorylates MLC at Ser19. The phospho-MLC mediates turning of myosin head, which generates the acto-myosin contraction and thus facilitates cell migration. However, hARD1 acetylates and inactivates MLCK stimulated by the Ca^2+^ signaling, which negatively control cell migration.

Initially, it was found that hARD1 and long MLCK interacted in the yeast two-hybrid system. This interaction was later confirmed in human cancer cells. hARD1 bound to and acetylated MLCK activated by Ca^2+^ signaling, and by so doing deactivated MLCK, which led to a reduction in the phosphorylation of MLC. Furthermore, Boyden chamber experiments showed that tumor cell migration and invasion were significantly inhibited by hARD1, and our mutation study revealed that hARD1 associated with an IgG-C2 motif of MLCK and acetylated the Lys608 residue in this motif. Moreover, K608A-mutated MLCK was not acetylated or inactivated by hARD1, and its stimulatory effects on migration and invasion were not inhibited by hARD1. Based on these results, we propose that hARD1 negatively regulates the MLCK-mediated migration and invasion of tumor cells (Fig. 7).

ARD1 functions as a catalytic subunit of the N-terminal protein acetyltransferase complex [20], [22], [28]. In addition, mouse and human ARD1s have been reported to participate in post-translational protein modification via ε-amino lysine acetylation, which is a common means of modifying proteins in eukaryotes. To date, only two proteins in mammalian cells have been found to be acetylated by ARD1; in particular, a mouse ARD1 variant (named mARD1^225^) was found to acetylate Lys532 in human HIF-1α [26]. However, since no equivalent of mARD1^225^ is expressed in human cells, it remains unclear whether HIF-1α is regulated by ARD1 in man [29]. Recently, we have reported that hARD1 directly acetylates β-catenin and augments β-catenin-dependent cell proliferation in non-small-cell lung cancer [27]. In the present study, long MLCK was found to be a novel hARD1-binding protein and to be acetylated by hARD1. Accordingly, long MLCK may be a third protein substrate of ARD1 in mammals.

Long MLCK is identical to short MLCK, except that it contains an N-terminal extension region, which contains six IgG-C2 motifs and two actin-binding domains. The IgG-C2 motif was originally identified to be an extracellular domain of adhesion molecules [30]. To date, this motif has been identified in a number of intracellular cytoskeletal proteins [31], and found to be involved in protein-protein interactions [6]. In addition, IgG-C2 motifs in long MLCK have many putative serine residues that could be phosphorylated by Ca^2+^ signaling [32]. Furthermore, the phosphorylations of IgG-C2 motifs activate MLCK, which in turn phosphorylates Ser19 of MLC, and in non-muscle cells, the phosphorylation of Ser19 of MLC has been found to be correlated with cell motility [33]. In the present study, hARD1 inhibited MLC phosphorylation by acetylating MLCK, and the hARD1-binding region on MLCK was found to be located between aa. 497 and 632 in its IgG-C2 region. Furthermore, Lys608 within this binding region was identified as the acetylation site. Thus, the IgG-C2 motif appears to play a critical role in long MLCK regulation by either phosphorylation or acetylation. In contrast, other transcripts of the *mylk1* gene (short MLCK and telokin) and skeletal muscle MLCK have no IgG-C2 motif, which encourages us to speculate that hARD1 only regulates long MLCK but that it does not control the contractions of skeletal or smooth muscle cells. hARD1 is expected to regulate cell behavior in association with the cytoskeletal system.

Ca^2+^/calmodulin-dependent activations of MLCK and MLC stimulate acto-myosin contraction, which promotes cell migration [34]. According to accepted theory, an increase in cytosolic Ca^2+^ concentration causes four calcium ions to bind to calmodulin, which allows calmodulin to enter the binding pocket at the MLCK C-terminus, and thereby, activates MLCK [35]. Accordingly, a high Ca^2+^ concentration should be maintained in moving cells, and in fact, it has been reported that moving cells show a 160–500% increase in Ca^2+^-dependent fluorescence intensity [36]. Thus, under these circumstances, MLCK and MLC are likely to be constantly activated, which poses the question … How do cells stop moving? Our findings suggest that hARD1 functions as such a brake, because hARD1 was found to deactivate MLCK and MLC specifically in the elevated Ca^2+^ state.

In addition to Ca^2+^/calmodulin, several protein kinases have been reported to regulate the enzymatic activity of MLCK. Long MLCK contains many consensus phosphorylation sites through its entire region, and the functional consequences of the phosphorylations of several of its serine/threonine residues have been well studied. In particular, the phosphorylation of one of two serine residues within its calmodulin-binding motif reduces its binding affinity for calmodulin, which reduces MLC phosphorylation. Some protein kinases have been reported to inhibit MLCK-mediated cell behavior by phosphorylating the sites; these include Ca^2+^/calmodulin-dependent protein kinase II, protein kinase A, protein kinase C, and p21-activated kinase [6], [37]. Furthermore, in addition to its calmodulin-binding and catalytic domains, two phosphorylation sites in long MLCK are targeted by members of the MAPK family, and the phosphorylations of these sites positively regulate MLCK by increasing its V_max_ [11]. In contrast to the known effects of MLCK phosphorylation, little is known about the effects of MLCK acetylation. In the present study, we found that hARD1 acetylates Lys608 of long MLCK and negatively regulates MLC phosphorylation by MLCK. To the best of our knowledge, this is the first report to claim that MLCK activity is regulated by lysine acetylation at the post-translational level.

Summarizing, we here identified a novel function of hARD1 in association with MLCK. Specifically, hARD1 was found to acetylate and deactivate Ca^2+^-activated MLCK, and this was found to lead to the dephosphorylation of MLC. Furthermore, this action of hARD1 was found to inhibit tumor cell migration and invasion. These results suggest that hARD1, a bona fide endogenous inhibitor of MLCK, can control tumor cell behavior. Accordingly, we suggest that additional studies be conducted on MLCK regulation by hARD1, because this study identifies hARD1 as a novel therapeutic target in the context of preventing tumor invasion and metastasis.

## Materials and Methods

### Reagents and antibodies

ML7, ML9, Y27632, ionomycin and BAPTA-AM were purchased from Calbiochem (La Jolla, CA), and phorbol myristate acetate (PMA), sodium butyrate (NaBu), and trichostatin-A (TSA) from Sigma-Aldrich (St Louis, MO). Anti-hARD1 antiserum was generated in rats against full-length hARD1 peptide [38]. Antibodies against GFP, phospho(Ser439)-MLCK, Myc-epitope, β-tubulin, and horseradish peroxidase-conjugated secondary antibodies were purchased from Santa Cruz Biotechnology (Santa Cruz, CA). Anti-acetyl-lysine, anti-MLC and anti-phospho(Ser19)-MLC were obtained from Cell Signaling Technology (Beverly, MA), anti-MLCK was from Sigma-Aldrich. Culture media and fetal calf serum were purchased from Invitrogen (Carlsbad, CA).

### Cell culture

HEK293 (human embryonic kidney) and HT1080 (human fibrosarcoma) cell-lines were obtained from the American Type Culture Collection (Manassas, VA). The cells were cultured in Dulbecco's modified Eagle's medium, supplemented with 10% heat-inactivated fetal bovine serum (FBS) in a 5% CO_2_ humidified atmosphere at 37°C.

### siRNAs and plasmids

The sequences targeting hARD1 (NCBI # NM_003491) corresponded to nucleotides 311–355 (si-ARD1), 366–392 (si-ARD1_2), and 528–554 (si-ARD1_3) of the coding region, and siRNA targeting green fluorescence protein was used as a control. cDNAs of hARD1 and long MLCK variant 1/2 (NM_053025, NM_053026) were cloned by reverse transcription and PCR using Pfu DNA polymerase, and the cDNAs obtained were inserted into pcDNA vector by blunt-end ligation. The plasmids for Myc-tagged MLCK fragments and Flag/SBP-tagged MLCK fragments were constructed by recombining PCR products with the corresponding plasmids. Point-mutations in MLCK and its fragments were made by substituting arginine for a lysine in MLCK, using a QuickChange^TM^ site-directed mutagenesis kit (Stratagene, Cedar Creek, TX).

### Immunoblotting and immunoprecipitation

Proteins obtained from cell extracts were separated by SDS/polyacrylamide gel electrophoresis (PAGE) and then transferred to Immobilon-P membranes (Millipore; Bedford, MA). Membranes were blocked with 5% nonfat milk, incubated overnight at 4°C with primary antibodies diluted 1∶1000–2000, and then incubated for 1 h with horseradish peroxidase-conjugated secondary antibodies. Antigen-antibody complexes were visualized using ECL-Plus (GE Healthcare, NJ). For immunoprecipitation, cell lysates were incubated with 5 µl of anti-hARD1 anti-GFP, anti-Myc, anti-MLCK antiserum, or preimmune serum at 4°C for 2 h. Immune complexes were further incubated with protein A/G-Sepharose beads (GE Healthcare) at 4°C for 4 h. Immunocomplexes were eluted by boiling for 10 min in a sample buffer containing 2% SDS and 10 mM dithiothreitol, subjected to SDS/PAGE and then immunoblotted using anti-hARD1, anti-MLCK, anti-p-MLCK, or anti-acetyl-lysine antibody.

### *In vitro* binding and acetylation assays

Myc-tagged or Flag/SBP-tagged MLCK peptides were expressed in HEK293 cells. Flag/SBP-tagged peptides were purified by Flag-affinity chromatography and eluted with Flag peptide. His-tagged hARD1 (His-hARD1) was expressed in *Escherichia coli* and purified by nickel-beads. For protein binding assays, mixtures of MLCK peptides (1 µg) and His-hARD1 (0.5 µg) were incubated at 4°C for 2 h, and protein interactions were identified by nickel-affinity chromatography and immunoblotting. For acetylation assays, 0.5 µg of His-hARD1 and 0.5 µg of MLCK peptides were incubated in a mixture of 50 mM Tris-HCl (pH 8.0), 0.1 mM EDTA, 1 mM DTT, 10 mM sodium butyrate, 20 µM acetyl-CoA, and 10% glycerol at 32°C for 4 h. Acetylated MLCK peptides were identified by immunoblotting with anti-acetyl-lysine antibody.

### Cell migration and invasion assays

For cell migration assays, transwell plates were purchased from Corning Life Science (Acton, MA). The lower chamber was filled with culture medium containing 10% FBS as a chemo-attractant. HT1080 cells (5×10^3^) in 100 µl of FBS-free medium were seeded into the upper chamber and incubated at 37°C in a 5% CO_2_ incubator for 6 h. For invasion assays, 0.5 mg/ml of Matrigel was loaded on the upper membrane surface. HT1080 cells (2×10^4^) in FBS-free medium were then seeded into the upper chamber and the lower chamber was filled with FBS-containing medium. Migrating cells on the lower membrane surface were stained with hematoxylin and eosin, and counted under an optical microscope at 40x.

### Statistical analyses

All data were analyzed using the unpaired Student *t-*test in Microsoft Excel 2003 (Redmond, WA). Results are expressed as means and standard deviations. Differences between experimental groups were considered statistically significant at the *p*<0.05 level. All statistical tests were 2-sided.

## Supporting Information

Figure S1 Cell viability was determined via MTT [3-(4,5-dimethylthiazol-2-yl)-2,5-dipheyltetrazolium bromide] assay. Cells were plated in 12-well plates and incubated with ML7 (20 microM), ML9 (20 microM), Y27632 (10 microM), Ionomycin (1 microM), PMA (30 nM), BAPTA/AM (20 microM), or DMSO vehicle in a 5% CO2 incubator for 16 h. After adding the MTT solution (Sigma-Aldrich) at 0.5 mg/ml, the plates were further incubated for 3 h. The establishing insoluble formazan was dissolved with 0.04 N HCl in isopropanol. Cell viability was determined by the differences in the absorbance at 570 nm using a spectrophotometer (Molecular devices corporation, CA).
(0.69 MB TIF)

Figure S2 Immunofluorescence staining of F-actin. After transfected with pcDNA (2 microg), phARD1 (2 microg), and siARD1 (80 nM), HT1080 cells were grown on coverslips and incubated in the presence of 20 microM of ML7, ML9, or DMSO for 16 h. The cells were washed twice with pre-warmed PBS and fixed in 3.7% formaldehyde in PBS at room temperature for 10 min. After three times washing with PBS, fixed cells were blocked with 1% bovine serum albumin in PBS for 30 min and then stained with Alexa Fluor 633 phalloidin for 10 min. Fluorescence images were observed using a confocal microscope (SZ40, Olympus, Japan).
(1.55 MB TIF)

Figure S3 Purity check of purified recombinant proteins. After purified through nickel- and Flag-affinity chromatography, His-tagged hARD1 (1 or 2 microg) and Flag/SBP-tagged MLCK_B (1 or 2 microg) were electrophoresed on SDS/polyacrylamide gels, and stained with Coomassie Brilliant Blue R-250. Protein band intensities were quantified using ImageJ 1.36b image analysis software (NIH, USA), and the protein purity was calculated by dividing the density of recombinant protein by total densities of stained proteins.
(1.60 MB TIF)
