# Supplementary figures and images for: Arrest Defective-1 Controls Tumor Cell Behavior by Acetylating Myosin Light Chain Kinase
Source: PLoS One. 2009 Oct 14;4(10):e7451. doi: 10.1371/journal.pone.0007451 (PMC2758594; doi:10.1371/journal.pone.0007451)

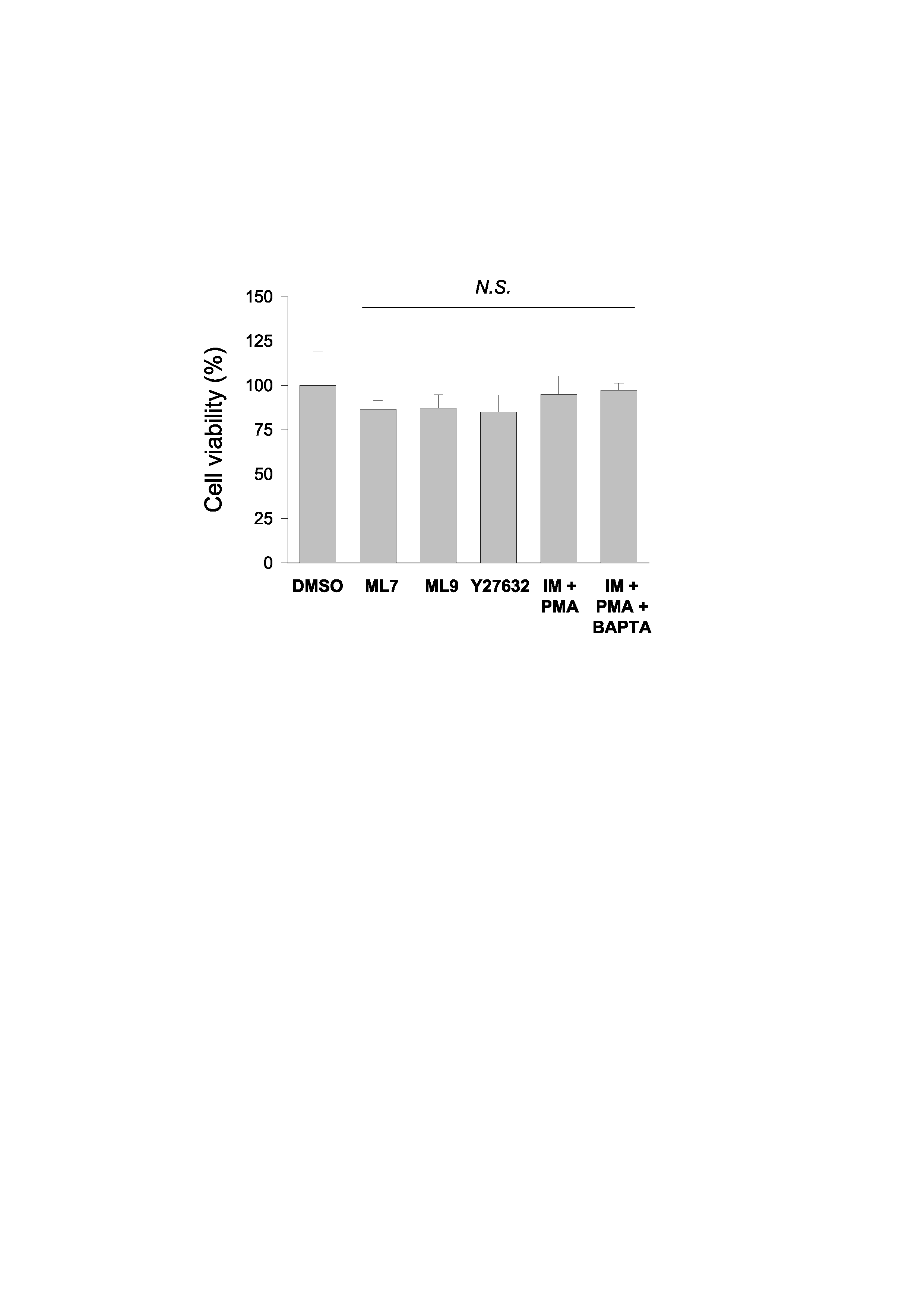

Supplement: Figure S1 — Cell viability was determined via MTT [3-(4,5-dimethylthiazol-2-yl)-2,5-dipheyltetrazolium bromide] assay. Cells were plated in 12-well plates and incubated with ML7 (20 microM), ML9 (20 microM), Y27632 (10 microM), Ionomycin (1 microM), PMA (30 nM), BAPTA/AM (20 microM), or DMSO vehicle in a 5% CO2 incubator for 16 h. After adding the MTT solution (Sigma-Aldrich) at 0.5 mg/ml, the plates were further incubated for 3 h. The establishing insoluble formazan was dissolved with 0.04 N HCl in isopropanol. Cell viability was determined by the differences in the absorbance at 570 nm using a spectrophotometer (Molecular devices corporation, CA). (0.69 MB TIF) [file pone.0007451.s001.tif]

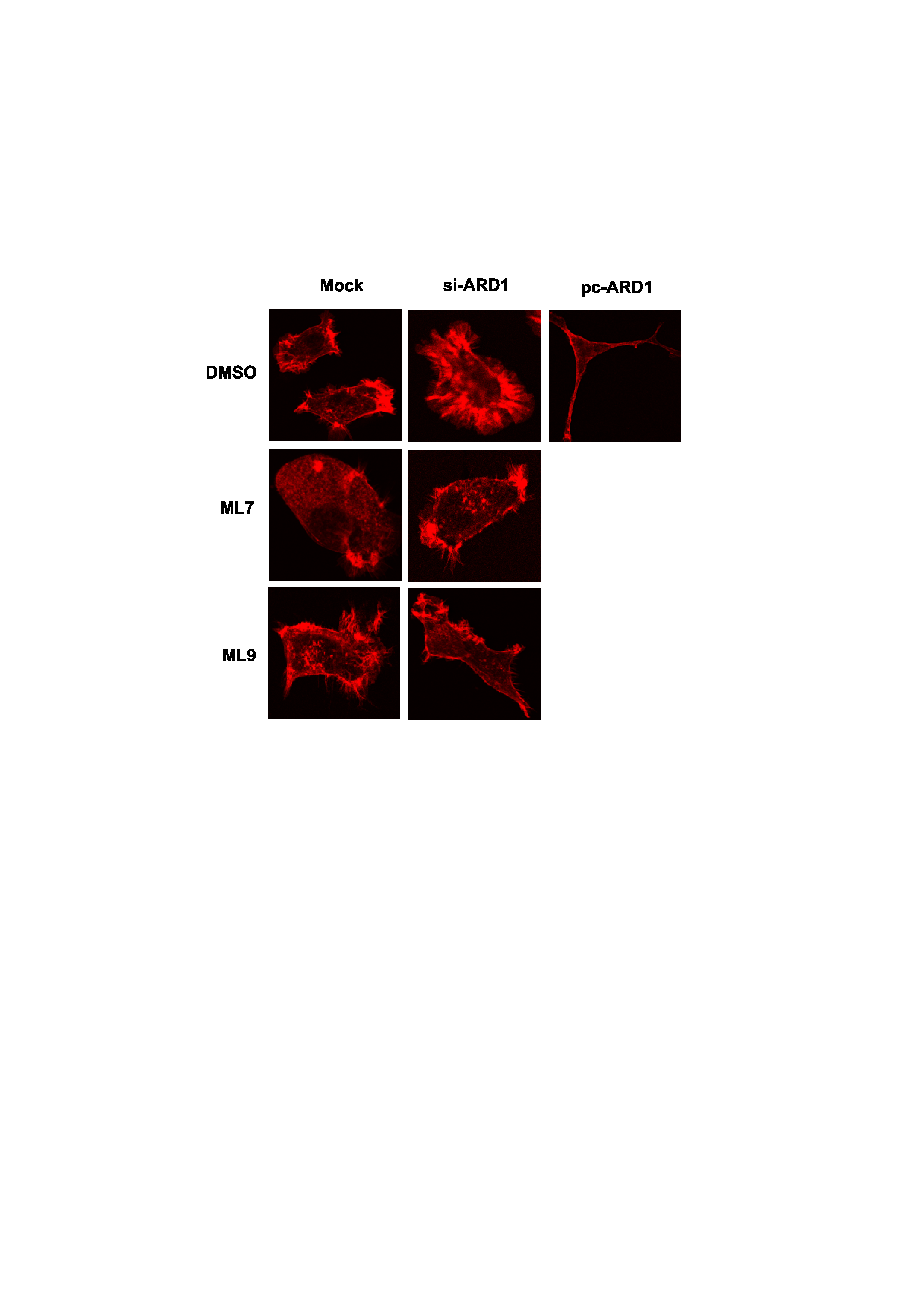

Supplement: Figure S2 — Immunofluorescence staining of F-actin. After transfected with pcDNA (2 microg), phARD1 (2 microg), and siARD1 (80 nM), HT1080 cells were grown on coverslips and incubated in the presence of 20 microM of ML7, ML9, or DMSO for 16 h. The cells were washed twice with pre-warmed PBS and fixed in 3.7% formaldehyde in PBS at room temperature for 10 min. After three times washing with PBS, fixed cells were blocked with 1% bovine serum albumin in PBS for 30 min and then stained with Alexa Fluor 633 phalloidin for 10 min. Fluorescence images were observed using a confocal microscope (SZ40, Olympus, Japan). (1.55 MB TIF) [file pone.0007451.s002.tif]

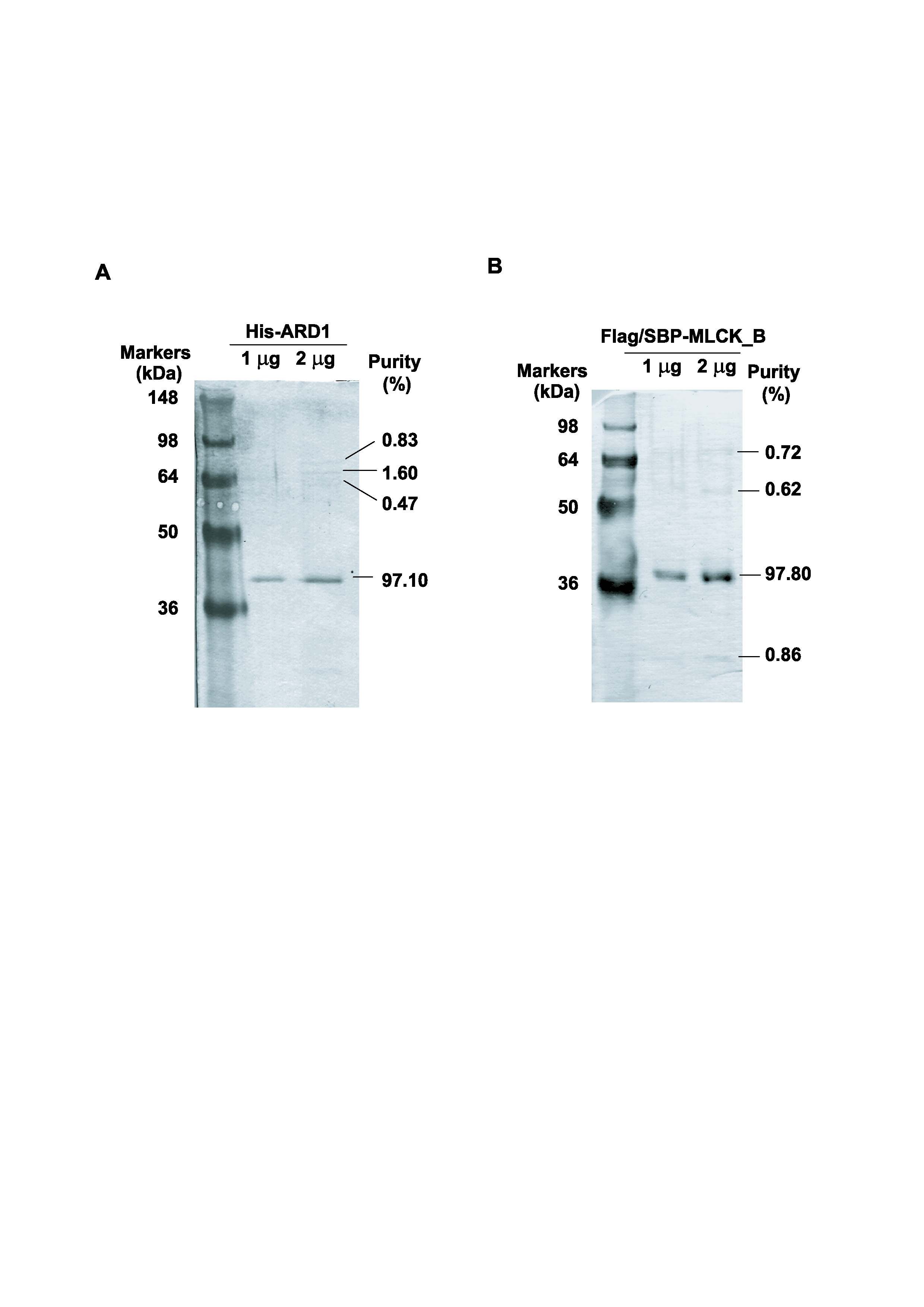

Supplement: Figure S3 — Purity check of purified recombinant proteins. After purified through nickel- and Flag-affinity chromatography, His-tagged hARD1 (1 or 2 microg) and Flag/SBP-tagged MLCK_B (1 or 2 microg) were electrophoresed on SDS/polyacrylamide gels, and stained with Coomassie Brilliant Blue R-250. Protein band intensities were quantified using ImageJ 1.36b image analysis software (NIH, USA), and the protein purity was calculated by dividing the density of recombinant protein by total densities of stained proteins. (1.60 MB TIF) [file pone.0007451.s003.tif]
